# A Modified Technique for Artificial Fusion in Unreconstructable Revision Total Knee Arthroplasty

**DOI:** 10.1016/j.artd.2021.06.003

**Published:** 2021-07-26

**Authors:** Mohammad Mahdi Sarzaeem, Moein Bonakdar, Keyvan Ramezani, Farzad Amouzadeh Omrani, Mohamadmahdi Omidian, S M Javad Mortazavi

**Affiliations:** a Department of Orthopedics, Imam Hossein Medical Center, Shahid Beheshti University of Medical Sciences, Tehran, Iran; b Department of Orthopaedic Surgery, Imam University Hospital, Tehran University of Medical Sciences, Tehran, Iran

**Keywords:** Revision TKA, Artificial arthrodesis, Conventional arthrodesis, Infected TKA, Limb length discrepancy

## Abstract

Knee arthrodesis is an acceptable treatment that leads to a stable joint with a lower rate of recurrence of infection in periprosthetic joint infections. One of the major problems in some revision cases is the bone loss that interferes with the bony union; therefore, some studies suggest artificial arthrodesis, which does not require bony union. The present descriptive retrospective study was conducted by reviewing the medical records of patients with periprosthetic joint infection complications. Patient satisfaction was evaluated after artificial arthrodesis, based on the visualized analog scale score and Oxford Knee Score. The mean Oxford Knee Score was 28, and the mean limb length discrepancy was 11 mm. In this new method, the length of hospitalization and leg length discrepancy was reduced, limb alignment and rotation was adjustable, and periprosthetic joint infection was controlled in nearly all patients.

## Introduction

Prosthetic joint infections (PJIs) occur in 1% of hip arthroplasties, 1%-2% of knee arthroplasties, and 25% of revision arthroplasties, every year [[1], [2], [3], [4], [5], [6], [7], [8], [9], [10], [11]] [4,12]. Infection costs show tremendous economic burden for tertiary-care centers and patients; in the United States, it was $566 million in 2009 alone, a number that is projected to reach $1.62 billion in 2020 [12].

Two-stage revision has the highest success rate, and hence, it is the most common operation for the management of PJI [13]. Owing to the high rates of recurrent infection and failure in revision TKA in medically compromised elderly patients, other treatment options including resection arthroplasty, arthrodesis, and above-knee amputation (AKA) are recommended [14]. Observations suggest that a patient who has experienced the results of resection arthroplasty and then was treated with arthrodesis is more likely to be acquiescent with the results of arthrodesis. Patients who underwent AKA for PJI reported a compromised ability to ambulate with a high mortality rate. In patients who were treated with AKA for PJI, a compromised ability to walk with a high mortality rate was observed [15].

Losing bone stock in multiple revision TKA is unavoidable; however, the bony union is needed for arthrodesis. Conventional arthrodesis is not successful in patients with significant bone loss mainly because of the great amount of shortening. In this case series, we aimed to evaluate the satisfaction of patients after artificial arthrodesis (our modified artificial arthrodesis method) based on VAS (visualized analog scale) score and Oxford Knee Score (OKS), operation time, limb alignment, and limb length discrepancy.

## Methods

The present descriptive retrospective study was conducted by assessing the medical records of patients referred to the Imam Hossein Hospital with PJI complication. A standard form was used to collect the personal and clinical information of the cases during admission. Patient satisfaction was evaluated after artificial arthrodesis, based on VAS (visualized analog scale) Score and OKS. The studied cases were not involved in the design of the research questions or assessment of the results; also, they had no information about the method or performance of the study. Patients were not requested to advise on elucidation or inscription of results. The information of patients remained confidential, and the outcomes were not published to studied individuals or the relevant patient communities.

### Patient history

**Figure 1:**
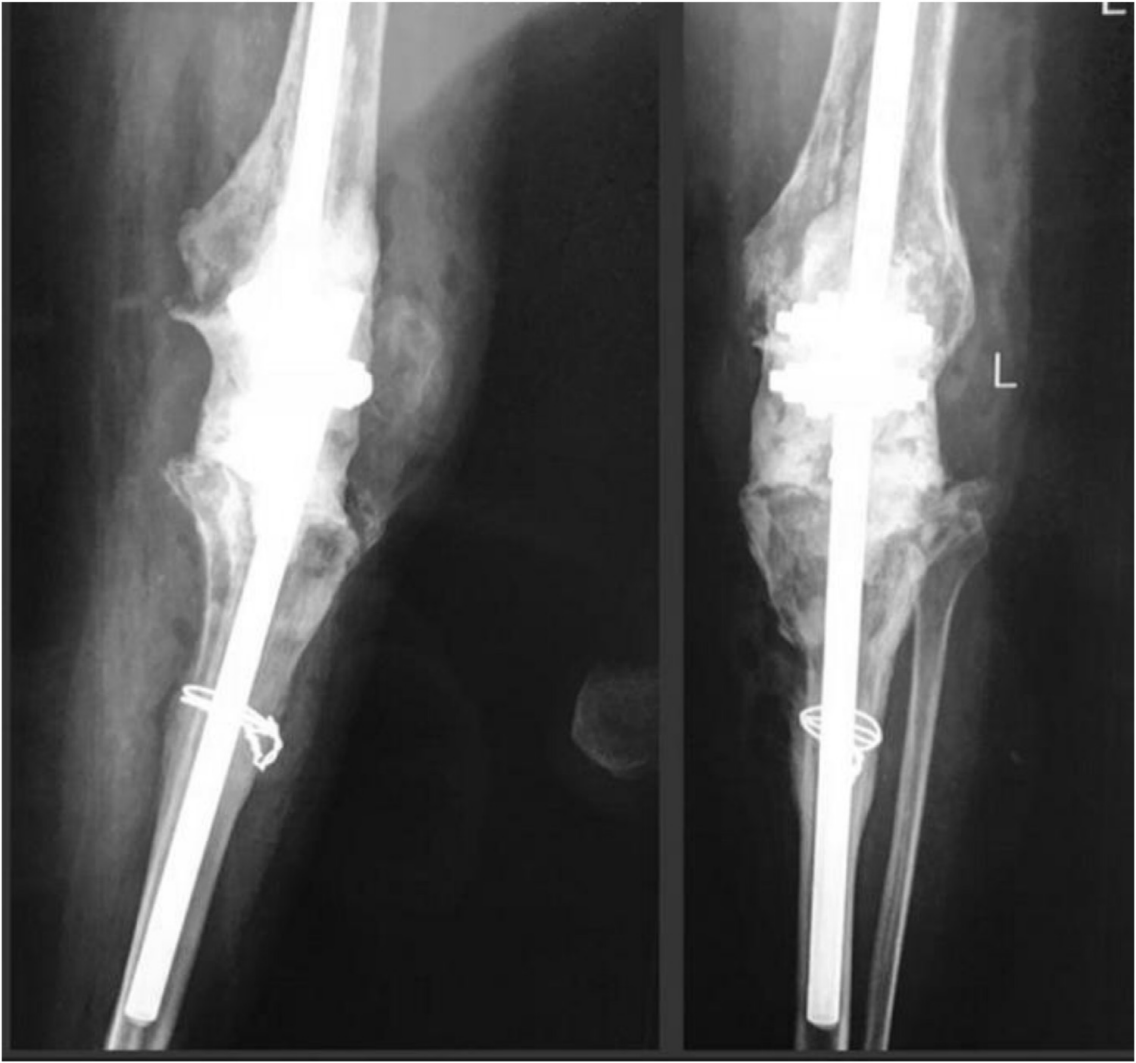
Anterioposterior and lateral postoperative knee radiograph. Patient with rheumatoid arthritis and osteoporosis.

In this series, 10 patients (3 males, 7 females) were described; they had a history of multiple revisions of TKA due to PJI. The first patient was a 71-year-old man, who suffered from severe ischemic heart disease and renal dysfunction and underwent 2 revisions before because of knee pain at rest and radiologic signs of loosening. The second patient was a 69-year-old female, who was a known case of uncontrolled long-standing rheumatoid arthritis and osteoporosis. (Fig. 1) She had been using immunosuppressive drugs and had PJI and radiologic signs of tibial and femoral component loosening.

**Figure 2:**
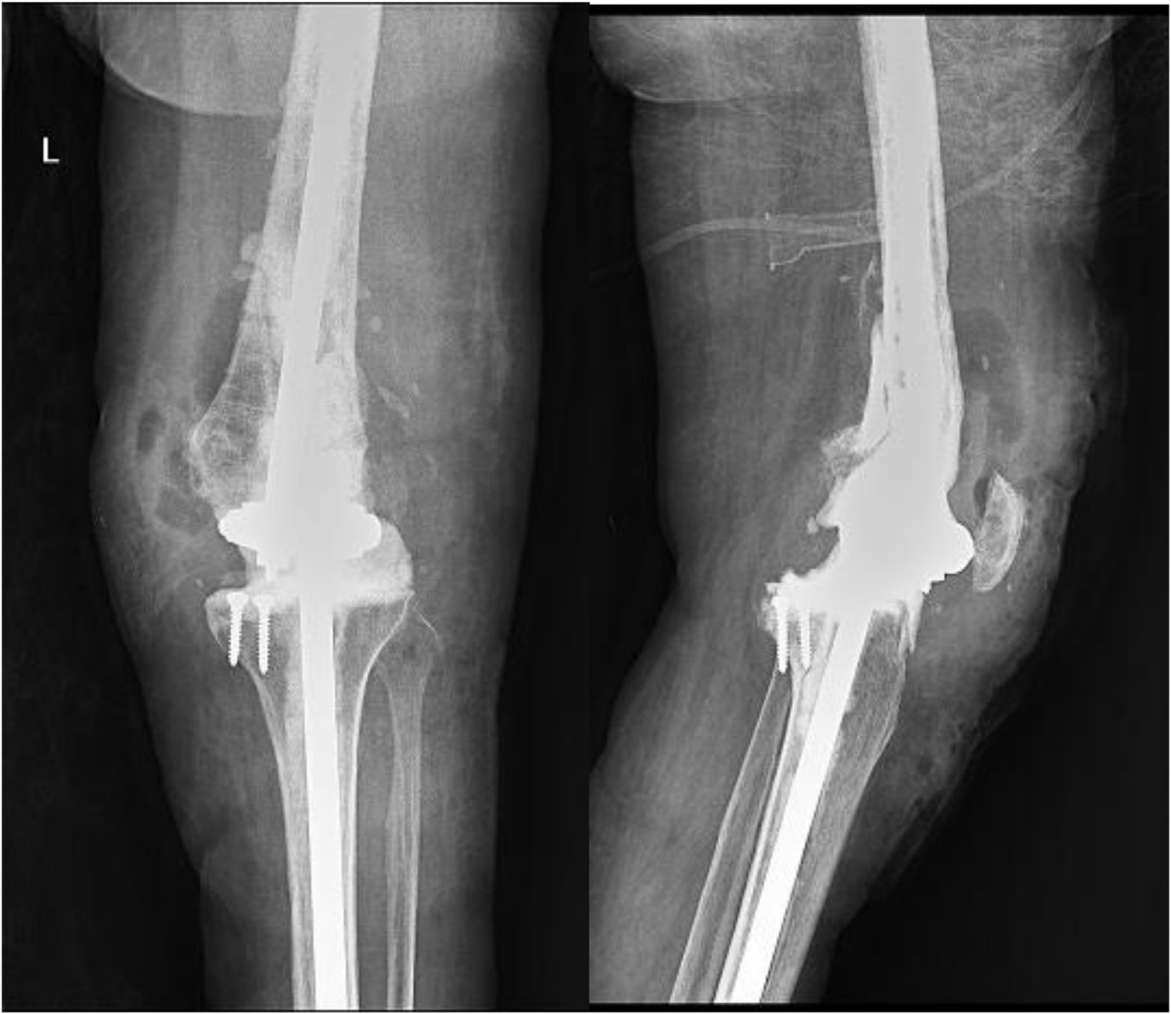
Anterioposterior and lateral postoperative knee radiograph. Patient with severe medial tibial plateau bone loss.

Third, fourth, fifth, and sixth patients suffered from severe pulmonary and cardiologic comorbidities. They had excessive debridement of necrotic and suspicious tissues, so artificial arthrodesis performed because of significant comorbidities. Seventh, eighth, and ninth (Fig. 2) patients suffered from chronic osteomyelitis, which was resistant to multiple irrigation and debridement, and long-term intravenous and oral antibiotic therapies; owing to prosthesis loosening, artificial arthrodesis was performed for them. The tenth patient was a woman with primary total knee arthroplasty who was referred to our clinic, and because of severe osteomyelitis and severe soft-tissue defect, the prosthesis was exposed; artificial arthrodesis was performed after massive debridement, but for soft-tissue management, the free muscular flap was needed, and the patient refused to follow the treatment and was satisfied with the outcome (Table 1).

**Table 1:** Descriptive summary of patient’s data after retrospective evaluation.

Patient ID	Age (y)	Sex	BMI (kg/m^2^)	Indication for RTKA	Microorganisms	Operation time (cutting/suture min])	Leg-length discrepancy (preoperative/postoperative)	VAS (preoperative)	VAS (postoperative)	Length of hospital stay (d)	Follow-up (wk)	OKS (preoperative)	OKS (postoperative)
1	71	M	29.23	Two-stage exchange (PJI)	MRSA	65	1/.9	7	1	5	115.28	15	26
2	69	F	37.68	Two-stage exchange (PJI)	MRSE	58	1.2/1	9	1	3	153.43	10	18
3	56	F	30.43	Two-stage exchange (PJI)	MRSA	73	1.8/2	6	1	21	28.46	14	35
4	67	M	27.91	Two-stage exchange (PJI)	*Escherichia coli* and *Enterococcus faecalis*	61	.9/.5	8	2	4	53.71	14	31
5	72	M	27.12	Two-stage exchange (PJI)	*Enterococcus faecalis*	72	.3/.5	8	1	2	112.46	11	28
6	66	M	34.40	Two-stage exchange (PJI)	Pseudomonas aeruginosa	70	1/.8	7	2	5	12.71	14	29
7	69	M	36.22	Two-stage exchange (PJI)	MRSA	64	.8/1.2	6	1	3	27.46	18	35
8	63	M	31.87	Two-stage exchange (PJI)	*Escherichia coli*	69	1.5/1	6	1	2	87.28	16	31
9	70	M	35.72	Two-stage exchange (PJI)	*Enterococcus faecalis*	61	2/1.5	8	1	5	65.46	13	22
10	65	F	26.65	(PJI and severe soft tissue defect)	MRSE	59	1.3/1.6	5	2	3	103.71	21	33
Average	66.8		31.72			65.2	.95/1.15	7	1.3	5.3	75.99	14.6	28.8

BMI, body mass index; RTKA, revision TKA; MRSA, Methicillin-resistant Staphylococcus Aureus; MRSE, Methicillin-Resistant Staphylococcus Epidermidis

### Surgical procedure

The patient was set in a supine position as for standard TKA, and an anterior midline skin incision was performed through the old scar. The operation included surgical debridement, removal of femoral and tibial components, and the bone cement. Samples were collected for microbiological culture and pathological assessment. Aggressive cutting was used to eliminate all residual cement, granulation and necrotic tissues, including in the suprapatellar pouch, medial and lateral gutter, and posterior section of the knee.

The sclerotic bony surfaces, which were located by femoral and tibial components, were cut back to the normal bone by using a high-speed tip burr. Irrigation of femoral and tibial canals was performed using 7-9 liters of normal saline with pulsed jet lavage. After complete debridement, tissue viability was specified by observing blood oozing from the soft tissue and remaining bone stock using 1-minute deflation of the tourniquet.

**Figure 3:**
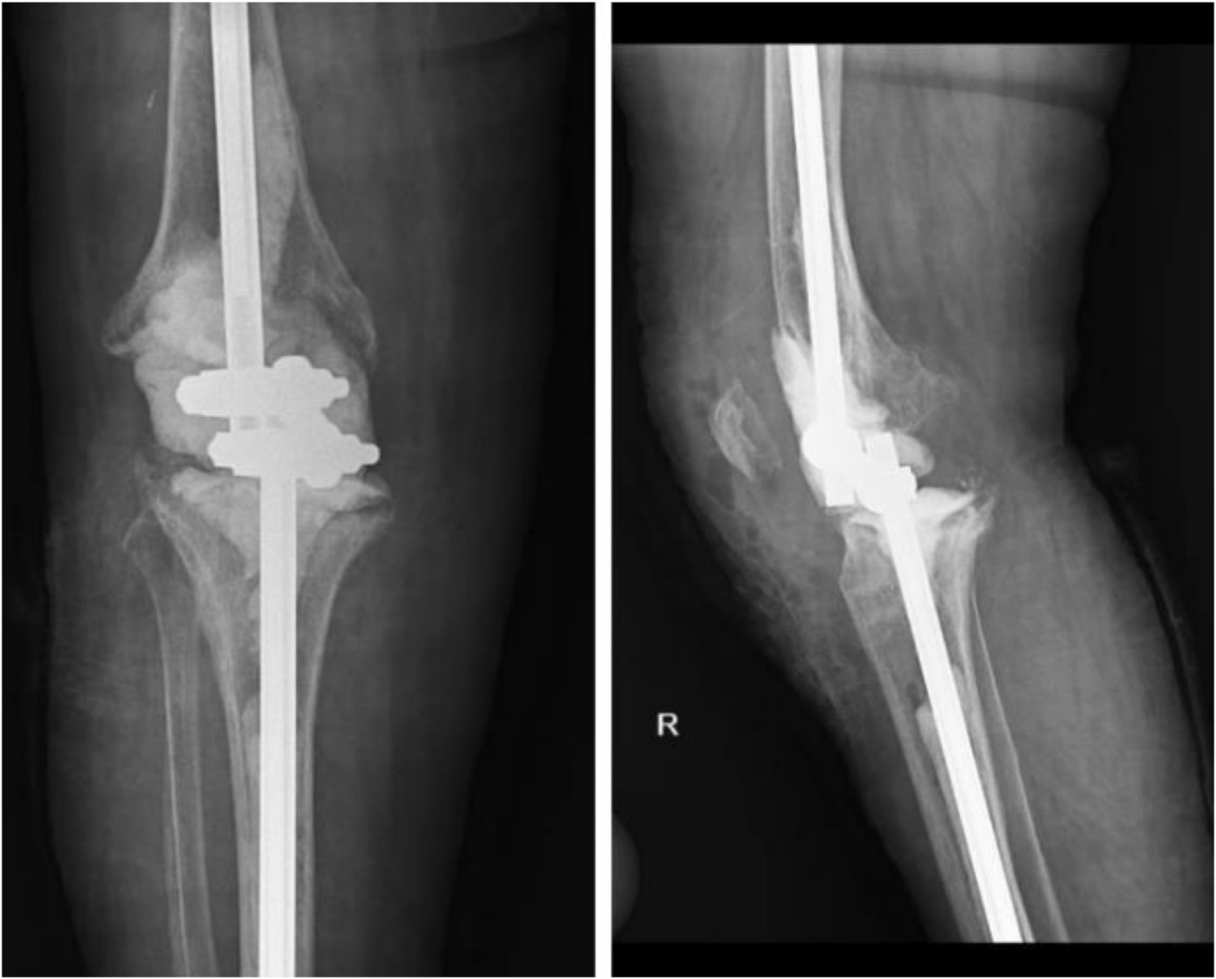
Anterioposterior and lateral postoperative knee radiograph.

Then, a 20-cm carbon fiber or steel rod of external fixator was inserted in tibial and femoral canals. These rods were filled with cement. Before inserting these rods, tibial and femoral canals were closed using 2 absorbable foams for pressurizing the cement, which would be injected into the canals in the next step. Tibial and femoral rods were connected to each other through connection clamps. Limb length and alignment, 5 degrees of valgus, 5-10 degrees of knee flexion, and neutral rotation were applied provisionally; then joint space was filled with cement (Fig. 3). After the operation, patients were permitted to bear weight as tolerated. Also, intravenous antibiotics were used based on microbiological culture and infection disease specialist suggestions.

## Results

The mean age of patients was 66.8 years. The mean duration of follow-up was 75.99 weeks. The mean duration of hospitalization was 5.3 days. No patient had surgical site discharge. After surgery, limb length discrepancy ranged from 5 mm to 20 mm (mean = 11.5 mm). Results of cultures were methicillin-resistant Staphylococcus aureus (MRSA) or methicillin-resistant Staphylococcus epidermidis (MRSE) in 5, *Enterococcus faecalis* in 2, *Pseudomonas aeruginosa* in 1, and *Escherichia coli* in 1 patient. One patient had *E. coli* and *E. faecalis* in culture, simultaneously.

After the surgery, VAS scores of patients decreased, and OKS increased. All fusions were clinically stable. Knee alignment was found to be near neutral in all cases, and all patients could walk at least 600 meters without any difficulty. Six patients could walk unlimited distances. Also, after the operation, one of our patients had an immense soft-tissue defect with exposed knee prosthesis; this patient underwent artificial knee fusion, but the soft-tissue defect remained untreated because the patient did not cooperate (Table 1).

## Discussion

The major result of this research was obtaining acceptable functional and VAS scores with a lower complication rate, after implementing this modified technique.

Knee arthrodesis is one of the main treatments for unreconstructable complicated total knee arthroplasty [4,6,16], and some studies reported a 50% to 80% success rate in different methods of arthrodesis [1,9]. The main target of arthrodesis is achieving bony union, which is challenging in patients with multiple revisions due to bone loss; it has been observed that arthrodesis in these patients leads to shortening from 2/5 to 6/9 cm [11], whereas more than 2 to 3 cm of shortening is an important factor that interferes with functional outcomes [6,16].

Artificial arthrodesis is an alternative method in these patients that can result in a stable knee without the need for bony union.

N. Hawi et al. studied artificial fusion by Link nail and antibiotic-loaded cement in 27 patients [5]. They had 4 cases of reinfection that one of them underwent knee amputation. They reported acceptable short form (SF)-36 and VAS scores at 36 and 67.1 months of follow-up, respectively [5].

Dae-Hee Lee et al. studied artificial fusion in 2 cases by a bundle of flexible rod and antibiotic-loaded cement [17,18]. They did not report rod failure in their follow-up, but the main concern in this method is rod failure that was reported to be 16% in a study by Capanna et al. on the treatment of around-knee tumors [17,18].

This modified technique has some advantages: External fixator rods and clamps are easily accessible and low cost; alignment and length were easily achieved by setting of connection clamps and rods; there is no need for bony union; this construct is strong and stable so allows the patient to walk full weight-bearing early postoperative; no failure was observed in follow-up, which shows the long-term strength and stability of this construct; there was no recurrence of infection in follow-up that may be due to the efficacy of antibiotic-loaded cement in this method; future conversion to arthroplasty can easily be achieved by extracting the cement, rods, and clamps; improvements of functional score and VAS score indicated the effectiveness of this method for satisfying patients.

One of the limitations of this study was the small number of cases. Another limitation was that a longer follow-up period is needed to evaluate long-term functional outcomes and possible failures; also, it would be better to compare this method with other methods in future studies.

Finally, this method may be a good alternative for fusion in unreconstructable complicated TKA, considering the acceptable functional and VAS scores with low complications.

## Conflicts of interest

The authors declare that they have no known competing financial interests or personal relationships that could have appeared to influence the work reported in this article.

## Appendix A Supplementary data

Conflict of Interest Statement for Omrani

Conflict of Interest Statement for Ramezani

Conflict of Interest Statement for Bonakdar

Conflict of Interest Statement for Mortazavi

Conflict of Interest Statement for Omidian

Conflict of Interest Statement for Sarzaeem
