# Supplementary material for: A Modified Technique for Artificial Fusion in Unreconstructable Revision Total Knee Arthroplasty
Source: Arthroplast Today. 2021 Jul 26;10:128–32. doi: 10.1016/j.artd.2021.06.003 (PMC8332371; doi:10.1016/j.artd.2021.06.003)
Supplement: Conflict of Interest Statement for Omidian [file mmc5.pdf]

# CONFLICT OF INTEREST STATEMENT

## *American Association of Hip and Knee Surgeons*

(Adopted from the American Academy of Orthopaedic Surgeons disclosure statement)

The following form **must be filled out completely and submitted by each author (example, 6 authors, 6 forms). All items require a response. If there is no relevant disclosure for a given item, enter "*None*."**

---

Manuscript Title

1. Royalties from a company or supplier (The following conflicts were disclosed) None

2. Speakers bureau/paid presentations for a company or supplier (The following conflicts were disclosed) None

3A. Paid employee for a company or supplier (The following conflicts were disclosed) None

3B. Paid consultant for a company or supplier (The following conflicts were disclosed) None

3C. Unpaid consultants for a company or supplier (The following conflicts were disclosed) None

4. Stock or stock options in a company or supplier (The following conflicts were disclosed) None

5. Research support from a company or supplier as a Principal Investigator (The following conflicts were disclosed) None

6. Other financial or material support from a company or supplier (The following conflicts were disclosed) None

7. Royalties, financial or material support from publishers (The following conflicts were disclosed) None

8. Medical/Orthopaedic publications editorial/governing board (The following conflicts were disclosed) None

9. Board member/committee appointments for a society (The following conflicts were disclosed) None

**Each author must sign AND print or type his/her name, date and submit a separate form**

In addition, one BLINDED Conflict of Interest form (no author names used) should be submitted per manuscript with all author disclosures.

---

Author Name Mohamadmahdi Omidian Author Signature Omidian Date 03/12/2021
